# The Impact of Job Burnout on Employees’ Safety Behavior Against the COVID-19 Pandemic: The Mediating Role of Psychological Contract

**DOI:** 10.3389/fpsyg.2022.618877

**Published:** 2022-02-24

**Authors:** Hui Liu, Yuexin Du, Huiwen Zhou

**Affiliations:** ^1^Institute for Human Resource Management, Zhejiang University of Finance and Economics, Hangzhou, China; ^2^School of Public Administration, Zhejiang University of Finance and Economics, Hangzhou, China

**Keywords:** job burnout, psychological contract, safety behavior, COVID-19, epidemic prevention

## Abstract

Employee safety behavior is critical for occupational health in work environments threatened by the COVID-19 pandemic. Meanwhile, the widespread and increasingly serious job burnout of employees is a complex and difficult problem for enterprises to handle during any epidemic. Therefore, it is helpful to identify and discuss job burnout and other main psychological factors that affect safety behavior to find appropriate solutions. Using the PLS-SEM method, the study explored the relationship between job burnout and safety behavior against the epidemic, as well as the mediating role of psychological contract. According to the local guidelines for controlling COVID-19, this study revised the safety behavior scale. Data were collected by structured questionnaires in May to July 2020 from Chinese employees (*N* = 353) who resumed their work after the outbreak of the pandemic. The findings confirmed that job burnout has a negative impact on safety behavior, and psychological contract play a partial mediating role in mitigating the negative impact. Specifically, the transaction dimension and relationship dimension of psychological contract negatively affected safety behavior while the development dimension of the psychological contract was not directly related to safety behavior. It is suggested that enterprises should take effective measures to reduce employees’ job burnout and implement flexible psychological contract management and intervention, so as to effectively improve the performance of work safety behavior. Based on the multidimensional model, the findings of this study shed light on promoting safety behavior to prevent the spread of epidemics.

## Introduction

With the worldwide spread of the COVID-19 pandemic and the dramatic economic policy response, the issues of restarting production have been rapidly brought to the fore. Many countries have stressed the need to restart much-needed business activities in response to the economic downturn brought about by the pandemic as the global economy seeks to develop smoothly and rapidly. However, this will undoubtedly lead to a large number of employees facing a high-risk and high mental stress (Rodríguez-Rey et al., 2020).

Therefore, it is helpful to identify and discuss the main psychological factors that affect safety behavior to find appropriate solutions (Canet-Juric et al., 2020). Because job burnout will affect the mental health and work behavior of employees, this has raised concerns not only for the possible aggravation of job burnout, but also for safety behavior performance during epidemics (Du and Liu, 2020; Gómez-Salgado et al., 2020).

In June 2020, China effectively contained the rapid expansion of COVID-19 cases. Prevention and control emergency response levels had been downgraded in all regions. The government emphasized that to do a good job of normalizing prevention and control, it is necessary to improve the detection ability, implement precise prevention and control, and ensure the resumption of work in an all-around way. With the gradual resumption of work and production, the rate of returning to work in large and medium-sized enterprises has reached 94%, close to the normal level in previous years.

During the same period, the epidemic situation in some countries entered the stage of a plateau or decline, and epidemic prevention measures such as “city closure” adopted by European countries have begun to show the effects. Many countries are also aware of the need to restart much-needed business activities. However, enterprises in various countries are also faced with the dual pressure of carrying out difficult daily production activities and seriously preventing epidemic situations.

China has accumulated unique experience in dealing with this dual pressure. All the enterprises that have resumed working in China have undertaken the major responsibility of their own epidemic prevention and attached great importance to it in their daily management. It is suggested that the enterprises that start operating again should establish good epidemic prevention work plans and epidemic prevention emergency plans, reserve necessary epidemic prevention materials, conduct epidemic prevention publicity and education, and determine and supervise the epidemic prevention responsibilities of each employee. According to the relevant epidemic prevention regulations of China, the enterprises involved in resuming work bear an important responsibility for the prevention of the epidemic. If any enterprise cannot control the epidemic situation in their workplace, it will be asked by the local health authorities to immediately stop work and the local health authorities will assist in handling the issue. Then, the enterprises involved cannot start work again for at least 2 weeks.

Against this background, many Chinese enterprises of all sizes have taken extensive measures to ensure that the health of their employees is not endangered. They suggested that employees should do the following their work and lives: conduct daily self-health monitoring; wear a mask when working and do not touch the inside and outside surfaces of the mask with your hands when you remove it; keep their office areas and rest areas tidy and clean their work stations, including their desks, armrests, seats, etc., at least once a day; take staggered meals and talk less when eating; avoid participating in gathering activities, and do not go to crowded or poorly ventilated places, such as restaurants, shopping malls, etc.; and wash your hands immediately after touching public facilities or others’ objects in the workplace.

To keep their jobs in the economic downturn, most Chinese workers who resumed work began their daily work under the threat of COVID-19. They cautiously abided by various epidemic prevention regulations and suffered more physical and mental pressure than usual. This will undoubtedly aggravate the job burnout of employees and expand the risk of the epidemic spreading, which will be a serious threat to the safety of employees, enterprises and societal health. Therefore, employee participation in safety behavior is of great significance for maintaining workplace safety and reducing the risk of COVID-19.

This paper establishes a theoretical model based on the PLS-SEM method and uses the relevant survey data of Chinese employees to study the relationship between job burnout and safety behavior in an epidemic environment and the impact of psychological contract on this relationship. The purpose of this study is to determine the mechanism of individual and organizational psychological factors influencing employees’ safety behavior and to provide a theoretical basis for improving employees’ safety behavior against COVID-19. In view of Chinese unique practice and experience in controlling the spread of COVID-19 and resuming production, the conclusions of this study will not only enrich the relevant theoretical system, but also provide beneficial management ideas and practical references for occupational health and safety management in other countries.

## Theoretical Analysis and Research Hypothesis

### Safety Behavior

Safety behavior has two dimensions: safety compliance and safety participation (Griffin and Neal, 2000). Safety compliance refers to the behavior of employees to ensure safety at work, such as complying with safety regulations and safety instructions. Safety participation is a kind of voluntary safety behavior beyond personal responsibility. For example, it includes proposing suggestions to improve safety and actively correcting the unsafe behaviors of colleagues.

Some literatures have studied of safety behavior from the perspective of personal characteristics. It was found that self-efficacy has a significant positive impact on safety compliance behavior and safety participation behavior, and perceived supervisor support positively moderates the relationship between self-efficacy and safety behavior (Zhang et al., 2018). Moreover, self-efficacy, safety attitude, safety awareness, safety knowledge and other factors are correlated with safety participation behavior and safety obedience behavior, among which self-efficacy has the greatest influence on safety participation behavior, and safety awareness has the greatest influence on safety obedience behavior (Zeng and Yang, 2016). Some previous studies have found that organizational behavior can affect employees’ safety behavior more widely (Wang et al., 2015). The results of an empirical study showed that the safety climate, work pressure, risk perception and safety management are significantly related to safety participation behavior and safety compliance behavior. Among them, the safety climate has the greatest impact on safety participation behavior, and risk perception has the least impact on safety compliance behavior (Wang and Yang, 2017).

Generally, safety behavior research is aimed at high-risk behaviors in some industries, such as the mining, transportation, smelting and chemical industries. Employee safety citizenship behavior is very important for workplace safety in high-risk industries. Wang et al. (2020) stated that it is necessary for managers to encourage employees’ safe citizenship behavior and to deal with stressful situations by strengthening safety trust intervention measures. To explore the safety behavior path, a theoretical model that was a function of both human resource management and psychological security was built for miners’ safety behavior (Wang et al., 2017). The results showed that psychological security has an obvious adjustment effect on safety behavior. In addition, the level of sense of psychological safety slowly increases the positive influence of safety compliance behavior and accelerates the reduction of the negative impact of safety participation behavior. The results also showed that wages, education and training have direct impacts on the psychological safety and safety compliance behavior of employees. From the perspective of interpersonal relationships, constructed a theoretical model of the relationship between the quality of employee relationships and the influence of safety behavior that takes job involvement as the mediating variable (Wang and Lu, 2019). The results showed that all dimensions of employee relationship quality in coal mining enterprises have a significant positive impact on safety compliance and participation behavior, among which satisfaction and trust have the most significant impacts. All dimensions of employee relationship quality have a positive impact on employee engagement, but the impacts of trust and commitment are obvious. Job involvement has a positive impact on safety compliance and participation behavior. Job involvement is a mediating variable between employee relationship quality and safety behavior. Construction workers are occupational groups with high risk and extreme mental stress, and their psychological capital will affect their mental health, job performance and safety behavior. He et al. (2019) found that the self-efficacy dimension of psychological capital has a positive impact on safety compliance and safety participation, and the resilience dimension has a positive impact on safety participation. In addition, communication competence plays a mediating role between the hope and optimism dimensions of psychological capital and safety participation.

In short, the concern and management of employees’ safety behavior in enterprises can effectively improve employees’ safety behavior and reduce the risk of harm. Safety behavior, as an action of employees that directly affects the workplace and their own safety at work, is the last link to determine the safety of enterprises, and will inevitably be influenced by many factors. However, when COVID-19 may threaten the production and service of enterprises for a long time, employees should always be alert to the threat of epidemic situation while ensuring normal work, which may lead to anxiety, depression, etc., and thus affect safety behavior. Therefore, under the background of epidemic prevention, it is particularly important to continuously improve employees’ safety behavior, and it is also necessary to explore whether there are certain antecedents that will affect employees’ safety behavior in epidemic prevention.

### Job Burnout and Safety Behavior

Job burnout is mainly reflectes in emotional exhaustion, depersonalization and low personal accomplishment, which will undoubtedly affect the personal behavioral effect of work. Resource conservation theory points out that employees who are burnout will consume more existing resources to adjust the fatigue, and if the resource consumption can’t be replenished in time, employees will fall into a vicious circle of insufficient resource supply, and employees will lack enough energy to work seriously, and even express their dissatisfaction through some negative attitudes or behaviors at work. Job burnout could result in a number of dysfunctional attitudes affecting individual employee behavior and organizational performance, including depression, negative orientations toward colleagues, reduced job performance, organizational commitment and job satisfaction (Kalliath, 2003; Swider and Zimmerman, 2010). Numerous studies have examined the common phenomenon and consequences of job burnout in the modern workplace (Jia, 2015; Liu et al., 2016; Sun et al., 2020). Therefore, what is the impact of job burnout in the workplace, and how does it specifically affect employee safety behavior?

Workplace stressors can lead to job burnout, and job burnout is a kind of psychological reaction to long-term exposure to stressors. Organizational change in the changing environment may form new work stressors, which will lead to negative health consequences for employees (Day et al., 2017; White et al., 2020). Chen and Ye (2020) constructed a conceptual model of young employees’ job burnout to clarify the influencing factors and structural dimensions of young employees’ job burnout. The conceptual model described the action paths of a combination of job burnout factors, revealed that the mismatch between individual factors and job situational factors is the root cause of job burnout. The occurrence or existence of factors that negatively affect the work environment and increase work pressure, and this will aggravate job burnout and affect the job performance of employees (Singh and Singh, 2018). Taking 120 nursing staff members as the research object, Li (2013) compared the influence of different degrees of job burnout on work efficiency. The results showed that the nursing quality, patient satisfaction and nurse satisfaction of the mild burnout group were significantly higher than those of the moderate burnout group and high burnout group, and nursing defects were significantly lower in the mild burnout group than those of the moderate burnout and high burnout groups. Furthermore, it was found that job burnout also affects employee safety behavior. Workload, management style and lack of work control have significant effects on psychological stress (Hao and Ye, 2020). Psychological stress also affects the safety compliance and safety participation of employees. Further research showed that psychological stress plays a significant mediating role in the relationship between job antecedents and safety compliance and safety participation (Wong and Chan, 2020).

In summary, job burnout has a negative impact on job engagement, work efficiency, safety compliance and safety participation. The current epidemic situation caused by COVID-19 is a serious threat to people’s health and life safety, which will undoubtedly increase the mental and physical burdens of employees, aggravate job burnout, and have adverse effects on mood and health, as well as negatively affect employees’ work behavior. Therefore, the following hypothesis was proposed:

**H1:** Job burnout negatively affects employees’ safety behavior during the COVID-19.

### Job Burnout and Psychological Contract

Job burnout not only affects the behavior of employees, but it also dynamically affects their work emotions. It also affects the various beliefs regarding the responsibilities and obligations of employees and organizations (Conway and Briner, 2002; Huang et al., 2014). Job burnout will reduce work ability and increase turnover intention, and work ability and turnover intention will affect job burnout. Turnover intention is positively correlated with emotional exhaustion and depersonalization of job burnout and negatively correlated with low personal accomplishment (Li et al., 2019). There is a significant negative effect of job stress on job engagement, and job burnout has a greater indirect impact on job engagement (Chambel and Oliveira-Cruz, 2010). Gao and Liu (2018) found that job burnout has a negative impact on job embedding and that subjective wellbeing has an intermediary role between them. Klein et al. (2020) found that there is a high correlation between work stressors and job burnout.

The concept of psychological contract emphasizes the existence and restriction of unwritten contracts and joint commitment between employers and employees. Most of the existing researches divided psychological contract into three dimensions: transaction, relationship and development. Transaction dimension emphasizes that employees expect economic returns such as salary and welfare, which is the comparative basic contract level; relationship dimension means that employees expect a good working atmosphere and harmonious interpersonal relationships in the organization, and they hope to be satisfied in the aspects of spiritual belonging and social interaction; development dimension is that employees want to get the promotion of positions, the promotion of professionalism or a bigger and better career development space, etc., which is a higher-level contractual pursuit. Because the three dimensions of psychological contract are generally progressive and have little relevance, they are generally discussed separately in research. However, besides the influence of the organization, the generation of psychological contract will also be influenced by the employees’ own emotions and state, and the job burnout caused by the high pressure of epidemic and severe situation during COVID-19 may make employees feel that the degree of achievement of psychological contract is not high, and even make employees neglect their attention to psychological contract. Supported by the above, the following were hypothesized:

**H2a:** Job burnout negatively affects the transaction dimension of psychological contract during COVID-19.

**H2b:** Job burnout negatively affects the relationship dimension of psychological contract during COVID-19.

**H2c:** Job burnout negatively affects the development dimension of psychological contract during COVID-19.

### Psychological Contract and Safety Behavior

Zhang and Li (2006) used the psychological contract framework to explore the fulfillment of the psychological contract and its consequences for organizational citizenship behavior. The analysis found that affective commitment plays a critical role in mediating psychological contract fulfillment, organizational citizenship behavior, etc. Zhang and Li (2017) found that the violation of psychological contract has a significant negative predictive effect on safety compliance behavior and safety participation behavior, and perceived organizational support plays a moderating role in the relationships between psychological contract violations and safety compliance behavior and safety participation behavior. Psychological security refers to the employees’ shared belief that the organization is a safe environment for members to express themselves without fear, embarrassment and punishment or other negative consequences in relation to their wellbeing, self-image and status (Edmondson, 1999). The sense of belonging, related to an individual’s experience of participating in a system or environment, makes people feel that they are an integral part of the environment or system. This concept embodies the sense of being valued, needed or accepted by a group, system or environment. Trust in organizational security has no direct and significant effect on security participation behavior (Conway and Coyle-Shapiro, 2012; Newaz et al., 2019), but psychological security and sense of belonging can mediate the relationship between trust in organizational security and security participation behavior (Liu et al., 2020).

Wang and Dong (2014) divided the formation process of psychological contract into three stages, including production, performance and realization; and established a relationship model between psychological contract and safety behavior. Enterprises could cultivate the safety behavior of employees by designing reasonable incentive mechanisms, conducting regular communication at the level of psychological contract and creating a safe and fair organizational atmosphere. According to the social exchange theory and the resource conservation theory, if the enterprise can guarantee the achievement of employees’ psychological contract, then employees will feel that they have been valued and given preferential treatment, and will have a sense of satisfaction and belonging psychologically. For the purpose of repaying the organization, they will abide by the regulations of the organization and take positive actions for the development of the organization, and they will also feel that they have more external and internal resources to work hard. In the environment of epidemic prevention, the achievement of employees’ psychological contract can make employees show more active safety behaviors to maintain the smooth operation of the organization, which is beneficial to the safety of production and service of enterprises. Supported by the above, the following were hypothesized:

**H3a:** The transaction dimension of psychological contract positively affects employees’ safety behavior during the COVID-19.

**H3b:** The relationship dimension of psychological contract positively affects employees’ safety behavior during the COVID-19.

**H3c:** The development dimension of psychological contract positively affects employees’ safety behavior during the COVID-19.

**FIGURE 1:**
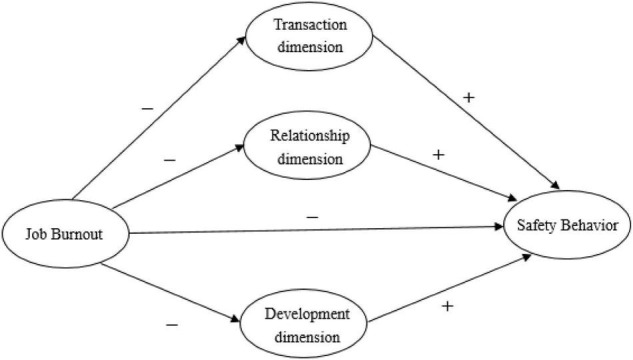
Research hypothesis model diagram.

Based on the above theoretical combing and research hypothesis, this study puts forward a research model of the relationship between related research variables under the epidemic background, as shown in Figure 1.

## Methodology

### Questionnaire Design and Collection

Demographic variables such as gender, age, educational background, the nature of the enterprise, industry, position, working years and time of starting work after the outbreak of COVID-19 in 2020 were selected as control variables. Questionnaire items of job burnout, psychological contract and safe behavior are all based on maturity scale. The job burnout scale developed by Li and Shi (2003) has been proven to have high reliability and validity. The reliability of each dimension of the original scale was above 0.8. This study used this scale for investigation, there are 15 items in the whole scale. Nine of them adopt positive scoring, such as “I believe that I can effectively complete the work”; and the other 6 items adopt the reverse scoring method, such as “Too much work makes me feel stressed.” Based on the psychological contract scale proposed by Chinese scholars (Zhang and Li, 2006; Sha, 2020), measured the variable in three dimensions, and the reliability of each dimension of the original scale was above 0.8. The scale consists of 13 items, all of which have positive scores. For example, “The company provides stable job security for employees.”

The scale of safe behavior referred to the scales used by Neal et al. (2000), discussed the items of the scale with three safety experts and two human resource management experts, and modified some items according to the requirements of COVID-19 epidemic prevention. It is subdivided into safety compliance behavior and safety participation behavior. The former includes the first to the fourth measurement items, and the latter includes the fifth to the ninth measurement items (Table 1).

**TABLE 1:** Safety behavior scale.

Dimensions	No.	Item
Safety compliance behavior	Q1	I have been keeping the epidemic prevention supplies in the right place.
	Q2	I have been using the anti-epidemic protective equipment as required.
	Q3	I have been abiding by professional epidemic prevention safety rules.
	Q4	In my work, I have been actively following the epidemic prevention arrangement of the management personnel.
Safety participation behavior	Q5	I have been trying my best to prevent the epidemic, whether there is supervision or not.
	Q6	I have been proposing suggestions to improve the epidemic prevention safety.
	Q7	I have been participating in team activities to improve epidemic safety.
	Q8	I have been willing to take the initiative to demonstrate standardized epidemic prevention methods to my colleagues.
	Q9	I have been taking the initiative to correct my colleagues’ incorrect epidemic prevention operations.

In this study, all items of three core variables were measured on a five-point Likert scale ranging from “1 = strongly disagree” to “5 = strongly agree.” From May to July in 2020, an anonymous questionnaire survey was conducted among enterprise employees who have returned to work in 17 cities of 6 provinces in China. Because the epidemic situation in China was basically controlled at this stage, and more effective methods of prevention and control of the epidemic situation in COVID-19 were found, the questionnaire collected can better reflect the situation of prevention and control of COVID-19 in most Chinese enterprises at that time. However, as the overall situation of prevention and control is still severe, researchers are affected by the prevention and control policies, and can’t visit enterprises too much. The questionnaire is collected mainly online and supplemented by offline. In this study, a total of 406 questionnaires were distributed, and finally 373 questionnaires were returned. After eliminating invalid questionnaires such as incomplete filling and identical filling contents, a total of 353 valid questionnaires were obtained for analysis.

### Statistical Methods

Because the purpose of this study is to explore the predictive ability of job burnout for prevention behavior through psychological contract and to provide theoretical guidance for the prevention of COVID-19, this study conducts exploratory research rather than pursuing the best model parameter estimation results. Compared with the covariance-based structural equation model, which has been widely accepted by researchers, the Partial Least Squares Structural Equation Model (PLS -SEM) has the advantages of being suitable for exploratory research to analyze complex models, extract orthogonal factors with zero correlation factors and predict dependent variables conveniently and flexibly (Sarstedt et al., 2014; Xiao, 2018). Therefore, this study uses Smart PLS 2.0 to construct the PLS-SEM to test the hypotheses proposed above (Davcik, 2014). It can directly obtain the *R*^2^ and explain the variance of the dependent variables to the maximum extent so as to enhance the accuracy of the exploratory research and data analysis.

In this paper, SPSS version 22 was used for the descriptive statistical analysis, group analysis and the common method bias, and the reliability and validity of the samples were analyzed by Smart PLS 2.0. Finally, the main effect and intermediary effect of this study were tested.

### Sample Analysis

Firstly, several important demographic variables are analyzed and summarized. Regarding the gender distribution, there were 155 males (43.9%) and 198 females (56.1%). The gender distribution is relatively balanced, which is in line with the high employment rate of Chinese women. Among the age groups, the most concentrated age group is from 21 to 50 years old, accounting for 94.6%, which showed that middle-aged and young people are the main employment groups during the epidemic. Because most of the subjects are front-line workers, few managers and technicians, most of the subjects have a low level of education. The specific data are shown in Table 2.

**TABLE 2:** Demographic analysis (*N* = 353).

Variable	Category	Frequency	Percentage
Gender	Male	155	43.9
	Female	198	56.1
Age	20 years old and below	9	2.5
	21–30 years old	136	38.5
	31–40 years old	120	34.0
	41–50 years old	78	22.1
	51 years old and above	10	2.8
Level of education	High school and below	231	65.4
	Junior college	65	18.4
	Undergraduate	53	15.0
	Master’s degree and above	4	1.1

The research also discusses the influence of demographic variables such as age, gender, enterprise nature and working years on the research variables in groups. The results show that there are significant differences in job burnout among employees of different ages, and three dimensions of psychological contract in working years. Other demographic variables have no significant influence on the research variables.

The significant *P*-value of age on job burnout is 0.012. The job burnout of employees aged 51 and above is significantly different from other age groups, and the order from high to low is 21–30 years old, 20 years old and below, 31–40 years old and 41–50 years old. It can be seen that in the process of epidemic prevention, older employees are more prone to job burnout than younger. Because faced with the great pressure of epidemic prevention, it will inevitably consume more physical and mental energy, and older employees can’t have enough energy and physical strength to adjust like young employees, which will easily lead to job burnout. The significant *P*-values of working years on transaction dimension, relationship dimension and development dimension are 0.035, 0.006 and 0.000, respectively. Employees who working more than 10 years are significantly different from other seniority levels, among which employees with 6–10 years have the largest gap, followed by less than 1 year and 1–5 years. This is also in line with the development process that employees’ psychological contracts gradually increase to the peak after they enter the organization, and then they are no longer full of expectations for the organizational contracts.

### Reliability and Validity Analysis

The common method bias of data will lead to a false relationship between research constructs. Harman’s single factor method suggested that after exploratory factor analysis, if the variance explanation of the first factor was more than 50%, the common method bias problem is not serious. In this study, SPSS 22.0 is used to conduct the overall exploratory factor analysis on the data of all items. The variance explained by the first factor was 47.577%, which was less than 50%, indicating that CMB was within the acceptable range.

The reliability of the questionnaire is mainly used to test the internal consistency of the results of the questionnaire measurement. In this paper, Cronbach’s alpha coefficient was used to measure the internal structure of the scale and the consistency of the measurement items. Cronbach’s alpha coefficient is acceptable as long as it reaches 0.70, 0.70–0.98 indicating high reliability, and below 0.35 indicating low reliability, which must be rejected. Then, the Kaiser-Meyer-Olkin (KMO) value is performed to determine whether the scale is suitable for factor analysis. When KMO value is greater than 0.9, it is very suitable; 0.7–0.8 means acceptable; below 0.6 is not suitable.

In this paper, the items of the safe behavior scale were reformed based on the epidemic situation, so the scale was specially tested. The results show that KMO = 0.912 and sig. = 0.00, which mean that the reliability of this scale is very high and very suitable for factor analysis. Then, we obtained the factor loading matrix of the scale after rotation, found that there are two factors in the safety behavior with clear structure, as shown in Table 3.

**TABLE 3:** Factor load matrix after rotation: safety behavior scale.

Dimensions	Items	Factor load after rotation		Variance interpretation rate
		Factor 1	Factor 2	
Safety compliance behavior	Q3	0.842	0.258	37.600%
	Q4	0.784	0.288	
	Q5	0.772	0.327	
	Q2	0.769	0.355	
	Q1	0.615	0.509	
Safety participation behavior	Q8	0.240	0.895	32.459%
	Q9	0.279	0.822	
	Q7	0.432	0.669	
	Q6	0.416	0.597	
Cumulative variance interpretation rate				70.059%

According to Table 3, factor 1 is the safety compliance dimension of safety behavior, including Q1, Q2, Q3, Q4, and Q5, although Q5 was originally assigned to factor 2; and factor 2 is the safety participation dimension of safety behavior, including Q6, Q7, Q8, and Q9. The loads of the two factors are greater than 0.5, and the cumulative variance that is explained by the factors is 70.059%, which verifies the two-dimensional division method of safety behavior adopted in this paper.

However, Davcik stated that the evaluation of Cronbach’s alpha coefficient is based on an impossible premise, that is, the reliability of all items is tau-equivalent; therefore, he suggested that researchers use the composite reliability (C.R.) instead, C.R. = (Σλ)^2^/((Σλ^2^) + Σδ). In general, the value of C.R. should be greater than 0.7 (Hair et al., 2010). Table 4 shows the C.R., standardized path load (β) of the reflection index and average variance extracted (AVE) of all latent variables. AVE is used to measure validity, the formula is AVE = (Σλ^2^)/n. The table shows that the standardized path load of all external models is greater than 0.7, and the C.R. is greater than 0.8; therefore, the reliability of the questionnaire is relatively ideal.

**TABLE 4:** SEM analysis (*N* = 353).

Contract	Item	Loading	C.R.	AVE
Job burnout	JB1	0.762	0.846	0.646
	JB2	0.847		
	JB3	0.800		
Transaction dimension	TD2	0.812	0.877	0.704
	TD3	0.854		
	TD4	0.851		
Relationship dimension	RD1	0.775	0.894	0.678
	RD2	0.872		
	RD3	0.811		
	RD4	0.833		
Development dimension	DD2	0.783	0.887	0.663
	DD3	0.807		
	DD4	0.824		
	DD5	0.843		
Safety behavior	PB1	0.939	0.930	0.868
	PB2	0.925		

*C.R. represents composite reliability; AVE represents average variance extracted; JB represents job burnout, TD represents transaction dimension, RD represents relationship dimension, DD represents development dimension, PB represents prevention behavior (the same as following tables).*

The convergent validity of latent variables can be evaluated by their factor loading, AVE and C.R. (Hair et al., 2010; Shiau and Luo, 2013). It can be seen from Table 4 that all the factor loadings in this study are greater than 0.7; each AVE of these facets is greater than the recommended threshold value of 0.5; and each C.R. is greater than 0.8. Therefore, the convergent validity was supported by the test.

Hair et al. (2012, 2013) suggests that there are two steps to test the discriminative validity of the model: first, test the cross loadings of the observed variables; second, the AVE square root of the latent variable in the first-order model is compared with its correlation coefficient with other latent variables. The results can be seen in Tables 5, 6.

**TABLE 5:** Cross loading of variables (*N* = 353).

	Job burnout	Transaction dimension	Relationship dimension	Development dimension	Safety behavior
JB1	0.762	–0.403	–0.370	–0.383	–0.373
JB2	0.847	–0.530	–0.431	–0.452	–0.445
JB3	0.800	–0.530	–0.500	–0.475	–0.567
TD2	–0.554	0.812	0.499	0.483	0.449
TD3	–0.482	0.854	0.511	0.523	0.483
TD4	–0.508	0.851	0.569	0.565	0.502
RD1	–0.393	0.467	0.775	0.451	0.420
RD2	–0.470	0.548	0.872	0.615	0.483
RD3	–0.432	0.508	0.811	0.666	0.491
RD4	–0.498	0.540	0.833	0.567	0.477
DD2	–0.413	0.427	0.540	0.783	0.441
DD3	–0.413	0.481	0.518	0.807	0.386
DD4	–0.475	0.556	0.584	0.824	0.475
DD5	–0.477	0.559	0.634	0.843	0.489
SB1	–0.574	0.559	0.566	0.514	0.939
SB2	–0.514	0.500	0.492	0.516	0.925

**TABLE 6:** Correlation coefficient and AVE square root between variables (*N* = 353).

	Job burnout	Transaction dimension	Relationship dimension	Development dimension	Safety behavior
Job burnout	0.804				
Transaction dimension	−0.614*	0.839			
Relationship dimension	−0.547*	0.628*	0.823		
Development dimension	−0.548*	0.624*	0.702*	0.814	
Safety Behavior	−0.585*	0.570*	0.569*	0.553*	0.932

The data in the diagonal of the matrix are the square root of AVE and the others are the corresponding correlation coefficients. *indicates that the correlation is significant at the significance level of 0.05.

As shown in Table 5, the reflected index loads of all latent variables are greater than the value when they interact with other latent variables. It can be preliminarily concluded that there is an ideal differentiation between the latent variables.

As shown in Table 6, the values on the diagonals in the table are greater than the absolute values on the corresponding horizontal and vertical columns. Therefore, it can be considered that each latent variable has good discriminant validity. From the statistical results in Table 6, it also can be seen that there is a significant correlation between the variables and their dimensions, which is pairwise correlation. Among them, job burnout has a significant negative correlation with the three dimensions of psychological contract and safety behavior, while the three dimensions of psychological contract have a significant positive correlation with safety behavior.

### Structural Model

This study used the path weighting scheme to estimate the standardized path coefficient of the model because the path weighting scheme can obtain the highest explanatory power. The coefficient of determination (*R*^2^) of safety behavior is 0.415. The analysis results of the path coefficient are shown in Table 7.

**TABLE 7:** Standardized path coefficient (*N* = 353).

Path	Path coefficients	S.E.	*T*-value
Job burnout - > Transaction dimension	–0.614	0.050	12.163***
Job burnout - > Relationship dimension	–0.547	0.051	10.724***
Job burnout - > Development dimension	–0.548	0.048	11.369***
Job burnout - > Safety behavior	–0.288	0.060	4.784***
Transaction dimension - > Safety behavior	0.178	0.060	2.962**
Relationship dimension - > Safety behavior	0.199	0.068	2.930**
Development dimension - > Safety behavior	0.144	0.074	1.955 N.S.

N.S. is not significant at the significance level of 0.5; ** and ***are significant at the significance level of 0.01 and 0.001, respectively. S.E. represents standard error.

First, the path coefficients of job burnout to the transaction dimension, relationship dimension, development dimension and safety behavior are significantly negative; therefore, H1, H2a, H2b, and H2c were verified. Second, the path coefficients of the transaction dimension and relationship dimension to safety behavior are positive and significant at the 0.01 significance level while the path coefficient of the development dimension to safety behavior is not significant. Therefore, H3a and H3b were verified, and H3c was not.

**FIGURE 2:**
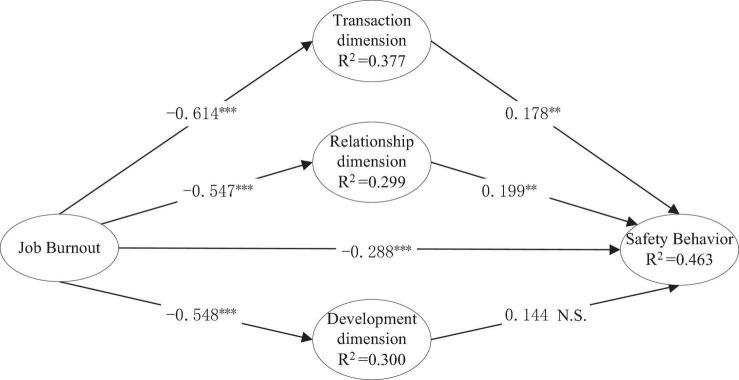
Influence path coefficient. *N*=353; N.S. is not significant at the significance level of 0.5; ** and ***are significant at the significance level of 0.01 and 0.001, respectively.

According to the previous path estimation results, only the significance of each path coefficient can be obtained. Whether the three dimensions of psychological contract can effectively mediate the effect of job burnout on COVID-19 safety behavior can be further tested. First, the effect of the development dimension on safety behavior is not significant, so the mediating effect of the development dimension on safety behavior and job burnout is not significant. Second, in other paths, whether the transaction dimension and relationship dimension effectively mediate the effect of job burnout and safety behavior cannot be concluded only by observing the above path coefficients, but rather they should be evaluated via calculations. According to the intermediary effect test process proposed by Zhao et al. (2010), PLS-SEM was used to analyze the output results, and the results are as follows: (1) The indirect effect of the transaction dimension on safety behavior (−0.614 * 0.178 = −0.109, *t* = 2.754) reached a significant level, indicating that there is a mediating effect; and the direct effect of job burnout on safety behavior was −0.288 (*t* = 4.949), reaching a significant level (see Figure 2), which indicating that there is a partial mediating effect. Because −0.614 * 0.178 * −0.288 > 0, it is a competitive intermediary effect. (2) The indirect effect of the relationship dimension on safety behavior (−0.547 *0.199 = −0.109, *t* = 2.778) is significant, which means that there is a mediating effect; and the direct effect of job burnout on safety behavior is −0.288 (*t* = 4.949), indicating that there is a partial mediating effect. Since −0.547 *0.199 * −0.288 > 0, it also indicates that it is a competitive intermediary effect. The specific data are shown in Table 8.

**TABLE 8:** Results of mediating effect (*N* = 353).

Indirect effect	Values	Standard deviation	*T*-values
Job burnout - > Transaction dimension - > Safety behavior	−0.109	0.040	2.754**
Job burnout - > Relationship dimension- > Safety behavior	−0.109	0.039	2.778**
Job burnout - > Development dimension - > Safety behavior	−0.079	0.041	1.922 N.S.

N.S. indicates that the effect is not significant at the significance level of 0.5. ** indicates that the effect is significant at the significance level of 0.01.

Under the specific background that COVID-19 mercilessly threatens people, the relationship among job burnout, psychological contract (transaction dimension, relationship dimension, and development dimension) and safety behavior was studied using the PLS-SEM. The χ^2^/df value of the structural equation model is 3.291 < 5, RMSEA value is 0.067 < 0.08, GFI = 0.876, CFI = 0.903, the values of IFI and TLI are all greater than 0.9. According to the output index of the model fitting, the fitting degree of the models is not very good, but overall, it is still in an acceptable range. The applicability and effectiveness of the job burnout—psychological contract—safety behavior model in the Chinese context was verified. The final causal model is shown in Figure 2.

According to the result of Figure 2, the main conclusions of this study are as follows: job burnout affects epidemic prevention safety behavior, and it is significant at 0.001 level, with an estimated value is −0.288. Job burnout negatively affects three dimensions of psychological contract: transaction dimension, relationship dimension and development dimension. The significance level is above 0.001, and its estimated values are −0.614, −0.547, and −0.548 respectively. The three dimensions of psychological contract have positive influence on epidemic prevention safety behavior, with estimated values of 0.178, 0.199, and 0.144 respectively. The significance level of transaction dimension and relationship dimension on epidemic prevention safety behavior is 0.005, but the development dimension has no significant influence on epidemic prevention safety behavior. Moreover, the transaction dimension and relationship dimension of psychological contract play a partial mediating role between job burnout and safe behavior, while the development dimension has no mediating role.

## Discussion and Conclusion

### Discussion

First, as shown in Figure 2, in the process of preventing and controlling COVID-19 in enterprises, job burnout accounted for 37.7, 29.9, and 30% of the three dimensions of psychological contract, namely, the transaction dimension, relationship dimension and development dimension, respectively. Job burnout is partially mediated by psychological contract, as well as directly influenced, and its explanatory ability to epidemiological prevention safety behavior is 46.3%. It is confirmed that job burnout negatively affects employees’ safety behavior in the process of preventing and controlling COVID-19 and producing, and psychological contract can play a part of intermediary role to alleviate this negative impact.

Second, during COVID-19, the severe situation of epidemic prevention and the unrelenting characteristics will increase the psychological pressure of employees, and it is easy for employees to get tired. Job burnout in this environment may cause employees to ignore some safety and epidemic prevention norms, thus showing behaviors that are not conducive to enterprise safety. These negative effects can easily cause human errors in work, relax the compliance with safety behavior standards, and eventually decrease the enterprise epidemic prevention effect. Therefore, the problem of job burnout during the epidemic period should be paid more attention and addressed better than usual.

Third, in the process of preventing COVID-19 in enterprises, the relationship dimension of psychological contract has the greatest mediating effect among the three dimensions, which indicates that in a public health crisis, the most important thing for a company is to treat each employee sincerely and respect employees. The transaction dimension is also very influential, which proves that if the company can provide competitive compensation and a safe working environment, it can also promote the safety behavior of employees in the process of epidemic prevention. In contrast, the development dimension usually focuses more on personal long-term growth and career development, training and promotion opportunities; therefore, it has no obvious relationship with the short-term epidemic prevention and emergency response behavior of employees. Thus, we should give full play to the positive role of psychological contract during the epidemic period. By improving the quality of the communication between leaders and subordinates in the company’s epidemic prevention management, organizations can provide effective situational support to reduce employees’ job burnout.

### Significance and Future Research

Based on the principles of applied psychology and the concept of safety behavior, this study constructed a theoretical framework and applied it to epidemic prevention and production safety, and the results provided occupational health countermeasures for enterprises to reduce the occurrence of epidemics.

This study makes several contributions. First, based on the unprecedented COVID-19 epidemic prevention background, we established a research model to observe and improve the safety behavior of employees. Second, according to the specific prevention and control behavior related to the epidemic, this study improved the existing safety behavior scale and provided a reference tool for similar research in the future. Third, studying the adverse effect of job burnout on employees’ safety behavior helps to understand the psychological mechanism of individual safety behavior. It is found that there are different impacts of the subdimensions of psychological contract on safety behavior, which indicate the complexity of the interactions among job burnout, psychological contract and safety behavior and highlights the necessity of multidimensional psychological contract intervention. Finally, the mediating role of psychological contract between job burnout and safety behavior provides the opportunity to better explain the psychological behavior process and reduce the risk of workplace epidemics.

More importantly, the research reported in this paper enriches the current body of knowledge by examining the impacts of job burnout on employees’ safety behavior and the mediating role of psychological contract. It provides theoretical guidance and practical suggestions for enterprises to establish appropriate strategies when a public health crisis occurs in the workplace.

However, there are some deficiencies in this study. First, since our cross-sectional study is based on survey data during a large-scale infectious disaster, the conclusions need to be confirmed by prospective cohort studies. Therefore, we suggest that a longitudinal design should be used in future studies to evaluate the research findings. Second, the universality of the conclusion needs further demonstration. Our investigation is limited to the specific period of China, and the revision of the safety behavior scale only referred to the epidemic prevention behavior of Chinese enterprises. In order to test the universality of the proposed model and the improved scale, future research should use employees from other countries and regions as samples for comparative analysis. Third, our model only studies the relationship between finite variables. Therefore, future research can study more variables in the context of public health safety and establish a variety of models involving multiple variables such as workplace epidemic risk, emotional stressors, organizational intervention, and proactive and prosocial safety behavior. Finally, since the three dimensions of psychological contract represent employees’ job pursuit and expectation at different levels respectively, this study adopts the way of sub-dimensions to make assumptions about this variable. Future research can explore the overall role of this variable to test the universality of the intermediary role of psychological contract under similar research background. And future research may focus on how the mental state of employees changes over time and how it affects safety behavior in epidemic prevention. Based on this dynamic relationship, safety management can promote flexible safety measures to elevate safety performance in the workplace.

## Conclusion

We must admit that widespread and increasingly serious job burnout is a complex and difficult problem for enterprises to address during any epidemic. This study explored the safety behavior mechanism of COVID-19 under the influence of two important psychological factors. The results of this study show that job burnout will negatively affect the work of employees and increase the possibility of unsafe behaviors among employees, which will have adverse impacts on the safety status of employees. However, in the process of preventing and controlling COVID-19 in enterprises, psychological contract can weaken the potential for unsafe behaviors by employees due to job burnout. If the organization attaches importance to the establishment of emotional trust and effectively connects employees with the organization, it can ensure the conscious and good implementation of epidemic prevention and safety behavior.

In order to address COVID-19 or other similar public health crises, enterprises can provide material resources and social support for employees to alleviate their job burnout. For example, different support projects and activities can be carried out to alleviate the negative emotions of employees, form an organizational atmosphere of humanistic care, and improve employees’ positive work emotions. This helps them to build a sense of trust and belonging at work and to better perform safe behavior.

## Data Availability Statement

The raw data supporting the conclusions of this article will be made available by the authors, without undue reservation.

## Author Contributions

HL, YD, and HZ conceived the idea, designed the study, and prepared the first draft and revised it. HZ and YD collected and analyzed the data. HL collected data and edited the original manuscript. All authors have approved the final version.

## Conflict of Interest

The authors declare that the research was conducted in the absence of any commercial or financial relationships that could be construed as a potential conflict of interest.

## Publisher’s Note

All claims expressed in this article are solely those of the authors and do not necessarily represent those of their affiliated organizations, or those of the publisher, the editors and the reviewers. Any product that may be evaluated in this article, or claim that may be made by its manufacturer, is not guaranteed or endorsed by the publisher.
